# PREventive Care Infrastructure based On Ubiquitous Sensing (PRECIOUS): A Study Protocol

**DOI:** 10.2196/resprot.6973

**Published:** 2017-05-31

**Authors:** Carmina Castellano-Tejedor, Jordi Moreno, Andrea Ciudin, Gemma Parramón, Pilar Lusilla-Palacios

**Affiliations:** ^1^ University Hospital Vall d’Hebron - Vall d'Hebron Research Institute Department of Psychiatry, CIBERSAM Autonomous University of Barcelona Barcelona Spain; ^2^ University Hospital Vall d’Hebron - Vall d'Hebron Research Institute Department of Endocrinology, Diabetes and Associated Metabolic Disorders (Ciberdem), Instituto de Salud Carlos III Autonomous University of Barcelona Barcelona Spain

**Keywords:** mHealth, motivational interviewing, physical activity, diet, sustained motivation, adherence

## Abstract

**Background:**

mHealth has experienced a huge growth during the last decade. It has been presented as a new and promising pathway to increase self-management of health and chronic conditions in several populations. One of the most prolific areas of mHealth has been healthy lifestyles promotion. However, few mobile apps have succeeded in engaging people and ensuring sustained use.

**Objective:**

This paper describes the pilot test protocol of the PReventive Care Infrastructure based on Ubiquitous Sensing (PRECIOUS) project, aimed at validating the PRECIOUS system with end users. This system includes, within a motivational framework, the Bodyguard2 sensor (accelerometer with heart rate monitoring) and the PRECIOUS app.

**Methods:**

This is a pilot experimental study targeting morbidly obese prediabetic patients who will be randomized to three conditions: (1) Group 1 - Control group (Treatment as usual with the endocrinologist and the nurse + Bodyguard2), (2) Group 2 - PRECIOUS system (Bodyguard2 + PRECIOUS app), and (3) Group 3 - PRECIOUS system (Bodyguard2 + PRECIOUS app + Motivational Interviewing). The duration of the study will be 3 months with scheduled follow-up appointments within the scope of the project at Weeks 3, 5, 8, and 12. During the study, several measures related to healthy lifestyles, weight management, and health-related quality of life will be collected to explore the effectiveness of PRECIOUS to foster behavior change, as well as user acceptance, usability, and satisfaction with the solution.

**Results:**

Because of the encouraging results shown in similar scientific work analyzing health apps acceptance in clinical settings, we expect patients to widely accept and express satisfaction with PRECIOUS. We also expect to find acceptable usability of the preventive health solution. The recruitment of the pilot study has concluded with the inclusion of 31 morbidly obese prediabetic patients. Results are expected to be available in mid-2017.

**Conclusions:**

Adopting and maintaining healthy habits may be challenging in people with chronic conditions who usually need regular support to ensure mid/long-term adherence to recommendations and behavior change. Thus, mHealth could become a powerful and efficient tool since it allows continuous communication with users and immediate feedback. The PRECIOUS system is an innovative preventive health care solution aimed at enhancing inner motivation from users to change their lifestyles and adopt healthier habits. PRECIOUS includes ubiquitous sensors and a scientifically grounded app to address three main components of health: physical activity, diet, and stress levels.

**Trial Registration:**

Clinicaltrials.gov NCT02818790; https://clinicaltrials.gov/ct2/show/NCT02818790 (Archived by WebCite at http://www.webcitation.org/6qfzdfMoU)

## Introduction

Information and communication technologies (ICT) applied to health and health care systems have been shown to increase their efficiency, improve quality of life, and unlock innovation in health markets. eHealth has emerged as a rapidly increasing area of promoting health, as well as bringing together end users and health care professionals to foster information exchange and effective communication. It is therefore the key technology for 21st century health care, as analyzed and projected within the European Commission eHealth Action Plan 2012-2020 [1]. The mHealth subdomain promises to bridge the gap between health settings and real-world scenarios [2]. Currently, mobile phones are widely used across developed countries. Their portability, ease of use, and ubiquity make them excellent tools to enhance the self-management of health and specifically chronic conditions.

The World Health Organization has estimated that in 2020 chronic or noncommunicable conditions accounted for 87% of deaths in high-income countries. Only 7% of deaths were attributed to communicable conditions and nutritional deﬁciencies and 6% to injuries [3]. The proportion of deaths worldwide caused by noncommunicable diseases is projected to rise from 59% in 2002 to 69% in 2030 [4]. Chronic diseases have traditionally included cardiovascular disease, diabetes, and asthma or chronic obstructive pulmonary disease. As treatments and survival rates have improved, chronic conditions now also include many varieties of cancer, human immunodeficiency virus and acquired immune deficiency syndrome, mental disorders (eg, depression, schizophrenia, and dementia), and disabilities such as visual impairment and joint disease. Many of these conditions are linked to an ageing society but also to lifestyle choices such as smoking, sexual behaviors, diet, and exercise, as well as to genetic predispositions. What these diseases have in common is their need for a long-term and complex response, coordinated by diﬀerent health professionals with access to the necessary drugs and equipment, and extending into social care.

For these reasons, mHealth is a key element to improving the management of chronic conditions because it includes a huge range of possibilities such as psycho-education, ecological momentary assessments, constant monitoring, reliable biofeedback, immediate tailored feedback, and brief interventions [5-8]. However, ensuring adherence and sustained motivation among users is still a complicated and unresolved problem as users have been frequently shown to lose interest in mHealth solutions resulting in decreased compliance of recommendations in the long run [9].

Health games or specialized motivating apps for mobile phones offer a possible solution to address the motivational factors involved in this problem. Thus, these apps try to bring empowerment, joy, engagement, and to offer a social connection to an otherwise rather dull self-management task [10-12]. As a result, the market for health-related games and apps has exploded in recent years, yielding over 100,000 apps in this area [1,2]. However, the information quality of the content of the apps is merely adequate [9]. In addition to functionality and esthetics, app developers should invest in providing content and quality behavioral tracking features, combined with a solid framework including motivational aspects. It is then recommended that they employ psychological techniques known to be effective in changing relevant behavior patterns and to improve the user experience and foster behavior change. Despite growing development and utilization of apps in research, additional experimental evaluation is required to understand whether the presence of particular content, behavior change techniques, motivational feedback, and so on, is associated with behavior change [9].

The PREventive Care Infrastructure based On Ubiquitous Sensing (PRECIOUS) project is a 7th Framework Programme for Research and Technological Development (FP7) project funded by the European Union and part of the eHealth and Ageing Initiative. Given increasing global obesity rates and physical inactivity, the project aims at preventing diseases such as type 2 diabetes and cardiovascular diseases by promoting healthier lifestyles. The project partners have designed and created a fully personalized system able to adapt to the user’s goals and preferences, combined with sensors (Bodyguard2, which is an accelerometer with 24h heart rate monitoring). Food intake, physical activity, stress levels, and sleep patterns are assessed in order to create a complete model of the user’s status called Virtual Individual Model, and subsequently certain measures and possibilities are suggested. A mobile phone‒based client app then finally delivers these interventions by running them within a privacy-ensuring sandbox, hence also facilitating developer’s efforts to intelligently integrate health data. A core concept in the PRECIOUS system is to operate under a common motivational umbrella guided by principles of motivational interviewing (MI) [13] in order to ensure long-term behavior change.

## Methods

### Objectives

The main goal of this pilot study is to assess users’ overall satisfaction, usability, and acceptability of the PRECIOUS system and to explore if MI is a feasible solution to foster adherence to PRECIOUS in a sample of end users. Moreover, we explore whether it triggers behavior change and builds up motivation to maintain sustained change towards healthy lifestyles.

### Hypothesis

Since this is a pilot study aimed at testing the feasibility of using the technology to achieve behavior change, no specific hypotheses have been put forward. Based on previous research on the efficacy of lifestyle apps and MI studies to foster behavior change [9,13-15], it can be expected that intervention groups (Groups 2 & 3) will show a tendency of change towards a healthier lifestyle compared to control group. These changes can be measured with the weight management questionnaires, the substance use/abuse questionnaires, and with several items related to the assessment of one of the main variables of the study (effectiveness).

No preliminary hypotheses have been established about possible differences between intervention Groups 2 and 3. Ideally, both groups will follow PRECIOUS recommendations with no significant differences between them. Thus, the potential benefits of the PRECIOUS app are expected to be achieved without the need for additional support (MI counseling). The PRECIOUS app’s continuous monitoring and feedback, as well as psycho-educational content and messages, are expected to effectively foster healthier lifestyles.

### Sample, Recruitment, and Study Design

A convenience sample (non-probabilistic) of patients has been recruited from a specialist outpatient consultation (anonymized for peer review). From an initial list of 55 potential participants, 24 (43.6%) were not included in the study because they were not interested or able to follow the full 3-month protocol. A total of 31 patients (71%, 22 women and 29%, 9 men) meeting the following inclusion criteria has been recruited: (1) patients under the care of medical specialists who adhere to national guidelines for morbid obesity and prediabetic condition following the American Diabetes Association criteria of fasting plasma glucose level from 5.6mmol/L (100 mg/dL) to 6.9mmol/L (125 mg/dL), (2) ˃18 years old, (3) body mass index ≥30, (4) understand and complete questionnaires in Spanish language, (5) use multimedia platforms such as personal digital assistants, tablets, laptops, mobile phones, or personal computers on a regular basis, and (6) have Android devices with an Internet connection. The rationale for choosing participants with Android devices is mainly technical because at this stage of the project, only an Android version of the app is available. Patients with the following criteria have been excluded from the study: (1) any mental (eg, cognitive impairment, severe psychopathology not stabilized) or physical condition that could interfere with the successful application of the research protocol, and (2) technological illiteracy or any other condition precluding the use of a mobile phone. Table 1 shows the demographic characteristics of the sample. Potential participants have been invited to take part in the study via telephone contact and have been randomly assigned into three groups:

- Group 1: Control group (treatment as usual + Bodyguard2)
- Group 2: PRECIOUS system (PRECIOUS app + Bodyguard2)
- Group 3: PRECIOUS system (PRECIOUS app + Bodyguard2 + MI)

**Table 1:** Demographic and health characteristics of the sample.

Characteristic	Group, mean (SD)			
	1 (n=11)	2 (n=10)	3 (n=10)	Total (n=31)
Age, years	37.45 (11.7)	41.9 (6.2)	38.9 (8.2)	39.35 (9)
Weight, kg	129.18 (17.5)	112.5 (11.7)	116.86 (21.3)	119.83 (18.2)
Height, cm	161.45 (9.3)	165.7 (5.5)	166.9 (9)	164.58 (8.3)
Body mass index, kg/m^2^	49.56 (5.6)	40.9 (2.8)	41.68 (4.4)	44.23 (5.9)
Maximum attained weight, kg	131.36 (19)	117.7 (11.5)	125.2 (20)	124.97 (17.7)
Number of diets last year	3.18 (4.2)	2 (3.2)	2.8 (3.1)	2.68 (3.5)

### Measures

Two types of measures will be registered: (1) A set of primary measures to evaluate users’ opinions and experiences with the PRECIOUS system, and (2) a set of secondary measures to explore the differences between groups across the sessions.

#### Primary Outcome Measures

To explore users’ acceptance of PRECIOUS system, the following aspects will be assessed:

Usability: 11-item questionnaire (10 items with 5-point Likert scale and 1 item with 10-point Likert scale) to assess users’ interaction with app and system interface.

Satisfaction: 14-item questionnaire (10-point Likert scale) to assess satisfaction with the different modules of the app (physical activity, diet diary), reports, feedback messages, and other interface elements.

Acceptance: 4-item questionnaire (10-point Likert scale) to measure the disposition to use and recommend the system to other patients.

Effectiveness: 15-item questionnaire (10-point Likert scale) to assess engagement, change, and adherence to healthy habits.

In Group 1 (control, treatment as usual), all these aspects will be assessed but with an adaptation of the same questionnaires based on their specific follow-up and the use of the Bodyguard2.

These measures were developed ad hoc for this study, but the usability items are based on the System Usability Scale [16] and acceptance and satisfaction items are based on the Questionnaire for User Interaction Satisfaction 7.0 [17].

#### Secondary Outcome Measures

Additional outcome measures will be included to explore the differences between groups across the sessions. Demographics and clinical data such as date of birth, gender, actual and maximum attained weight, height, number of diets in previous year, and questions related to self-esteem will be registered.

Health-related quality of life will be assessed with the Spanish version of the Short Form (SF)-12v2 Health Survey [18,19], a shorter version of the SF-36v2 Health Survey that consisted of 12 questions (3 and 5-point Likert scale) to measure functional health and well-being from the patient’s point of view. The SF-12v2 covers the same 8 health domains as the SF-36v2: physical functioning, role-physical, bodily pain, general health, vitality, social functioning, role-emotional, and mental health.

Weight management will be assessed with Spanish versions of S-Weight and P-Weight questionnaires [20,21]. P-Weight is a 34-item questionnaire (5-point Likert scale) developed to assess the process of change in weight management. S-Weight consists of 5 mutually exclusive items that allow allocation of participants to one of the five stages of change of the Transtheoretical Model [22] for weight management: precontemplation (not ready), contemplation (getting ready), preparation (ready), action, and maintenance.

The symptoms of depression, anxiety, and stress will be measured with the Spanish version of the Depression, Anxiety, and Stress Scale (DASS) [23]. DASS is a 21-item questionnaire (4-point Likert scale) developed to assess the severity of the core symptoms of depression, anxiety, and stress, and it is not designed as a diagnostic tool. For each scale, it offers a severity rating: normal, mild, moderate, severe, and extremely severe.

Alcohol consumption will be measured with the Spanish version of Alcohol Use Disorders Identification [24,25], a 10-item questionnaire (4-point Likert scale) developed by the World Health Organization as a simple method of screening for excessive drinking and to assist in brief assessment.

Tobacco consumption will be measured with the Spanish version of Fagerstrom Test for Nicotine Dependence [26,27]. It contains 6 items (some with a yes/no response and some with a 4-point Likert scale) that evaluate the quantity of cigarette consumption, the compulsion to use, and dependence.

Sleep quality will be assessed with the Spanish version of the Pittsburgh Sleep Quality Index [28,29], which measures sleep quality and disturbances over a 1-month time interval with 19 items. It assesses the following components: subjective sleep quality, sleep latency, sleep duration, habitual sleep efficiency, sleep disturbances, use of sleeping medication, and daytime dysfunction.

Physical activity will be assessed with the PRECIOUS app in the intervention groups (Groups 2 and 3). Physical activity will be measured with accelerometer data from the patient’s mobile phone. The accelerometer counts the steps walked and provides the patient a graphic result with total accumulated steps and time spent. If the patient cannot wear the mobile phone, the PRECIOUS app allows them to manually introduce the activity performed and it calculates the equivalence in steps.

Nutritional habits will be assessed with the PRECIOUS app in the intervention groups (Groups 2 and 3) with a diary that allows the patient to register the different meals each day.

**Figure 1:**
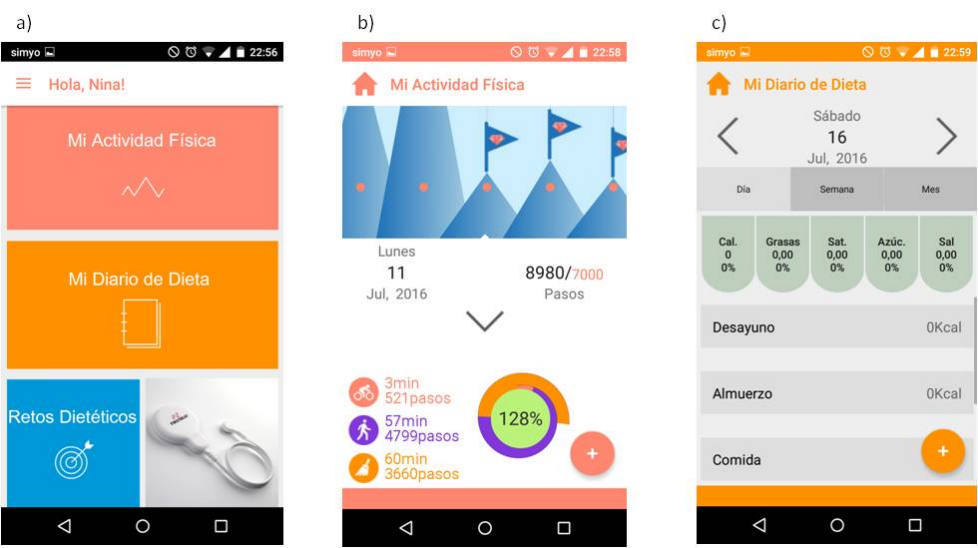
Screenshots of PRECIOUS app: a) home screen, b) physical activity app, c) dietary app.

The PRECIOUS app architecture and the interface of the physical activity and nutritional habits modules includes the four processes of MI (engaging, focusing, evoking, and planning) and MI micro-abilities (open questions, affirmations, reflections, summaries, and offering information to the users with their permission). Figure 1 shows screenshots of the PRECIOUS app. For the control group (Group 1), physical activity and nutritional habits will be assessed with self-reports using a weekly diary on paper. A total of 6 weeks will be registered: 3 weeks at the start of the study and 3 weeks at the end of the study.

To measure cardiovascular activity, the Bodyguard2 (Firstbeat) device will be used. This device works as a Holter measurement and allows recording of 24-hour periods. The Bodyguard2 is a lightweight and unobtrusive device, attached to the chest with two adhesive electrodes, and is reliable for recording heart rate variability (R-R interval) and movement data. A total of 6 days/nights will be registered in the groups: 3 days/nights at the start of the study after the first session (week 1) and 3 days/nights before the last session (week 12). Data collected with Bodyguard2 device will be analyzed with an adaptation of the Lifestyle Assessment Analysis Server Software (Firstbeat) performed by the PRECIOUS consortium partners (Aalto). This software provides a detailed report of the measured period: stress levels, recovery reactions, physical activity, exercise, sleep quality, and expended kilocalories. At the follow-up assessments (Sessions 3 and 5), patients will receive a report accompanied by oral explanations of the results provided by the researcher in charge of the follow-up.

### Procedure

**Figure 2:**
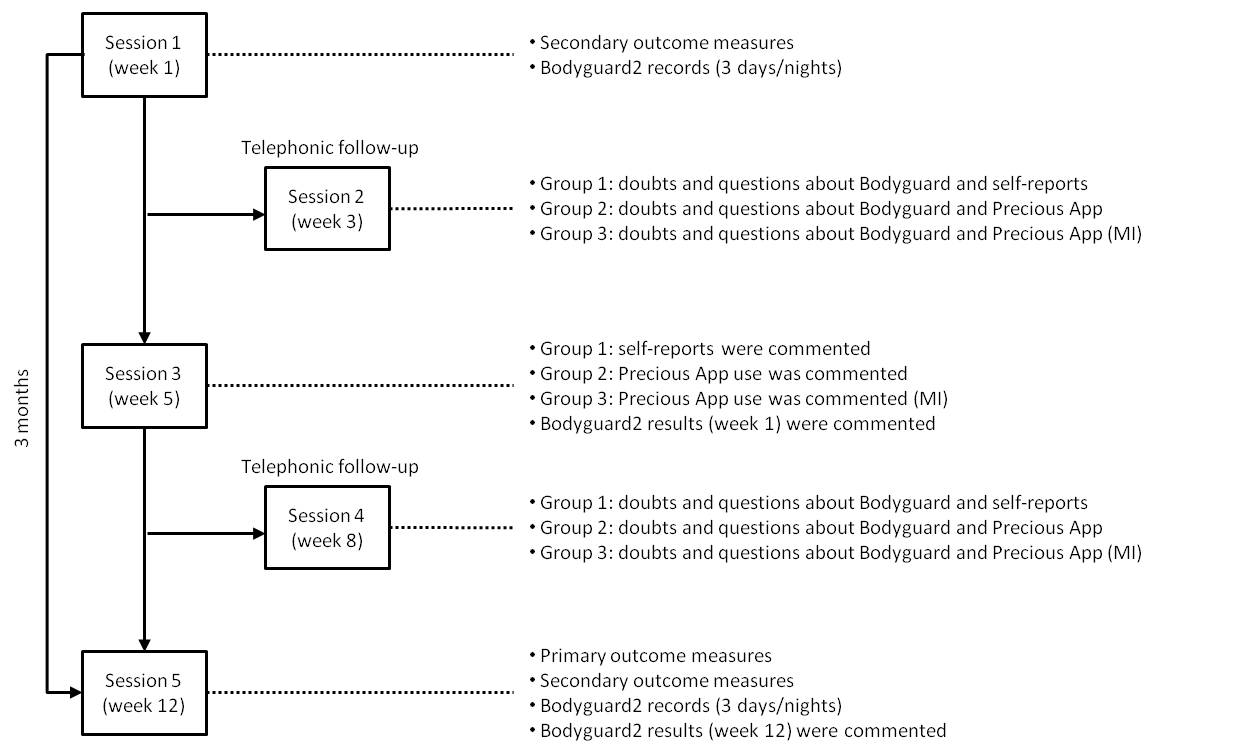
Flowchart of sessions during the pilot test and the measures assessed in each one.

The study will have a duration of 3 months with the following scheduled follow-up appointments: Session 1 (Week 1) as a baseline, Session 2 (Week 3), Session 3 (Week 5), Session 4 (Week 8), and Session 5 (Week 12). Figure 2 shows a detailed timeline of the procedure and measures assessed in each session. In Session 1, patients will receive a detailed explanation of the study, will be provided with written and oral information, and will give informed consent. They will then answer all of secondary measures in a semistructured interview and then receive the Bodyguard2 device. Patients from the intervention groups (Groups 2 and 3) will be invited to download the PRECIOUS app on their mobile phone and create a user account. Patients from the control group (Group 1) will receive a 3-week paper diary to register physical activity and nutritional habits. They will also follow their treatment as usual for morbid obesity. Specifically, treatment as usual consists of regular follow-ups with the endocrinologist and standard routine tests (blood samples, glucose) and anthropometric measurements carried out by a nurse. Session 1 lasts 1 hour approximately. Sessions 2 and 4 will be telephone follow-ups, lasting a maximum of 10 minutes. Only doubts and questions regarding PRECIOUS will be addressed (specific measures not collected) in telephone follow-ups. In all three groups, the use of the Bodyguard2 device during the 3 days/nights after Session 1 will be verified. In these telephone follow-ups, the researchers and users agree on the day of the next session in the hospital. Session 3 will be focused on discussing the results obtained with Bodyguard2 (only in Groups 2 and 3), to understand users’ preliminary opinions about the PRECIOUS app. In Group 1, the weekly self-reports of physical activity and nutritional habits will be discussed. Session 3 lasts 1 hour approximately. Session 5 will be the last of the study. Primary and secondary measures will be answered, and the results obtained with Bodyguard2 will be discussed. This last session lasts 1 hour and 30 minutes approximately. All the Group 3 follow-ups will be performed following MI principles. In Groups 1 and 2, MI principles will not be applied. MI has the main goal of getting participants to resolve their ambivalence about changing behavior while not evoking resistance. For such purposes, a directive, person-centered counseling style is employed. This approach is intended to elicit behavior change by helping patients explore and resolve ambivalences and understand the discrepancy between participants’ current behaviors in terms of lifestyles and desired goals [13]. The researcher in charge of these groups has specific training to lead MI sessions.

### Statistical Plan

The statistical analysis will consist of intergroup (Groups 2 and 3) descriptive and mean comparison analyses concerning primary outcome measures (usability, user satisfaction, acceptance, and system effectiveness) and overall assessment of the motivational aspects of PRECIOUS. In our pilot test, the method to assess minimal clinically important difference will be employed (distribution-based method, ie, ±1 mean standard deviation) to study differences between groups related to the main primary outcomes [30].

Secondary analyses will describe and explore changes between groups (inter and intra) in the following secondary outcome measures: (1) health-related quality of life using SF12v2), (2) weight management using S-Weight & P-Weight, (3) severity of the core symptoms of depression, anxiety, and stress using DASS, (4) alcohol consumption using Alcohol Use Disorders Identification test, (5) tobacco use with the Fagerstrom Test for Nicotine Dependence, and (6) sleep quality using the Pittsburgh Quality of Sleep Index. For these purposes, correlational, median comparisons, and nonparametric tests (chi-square tests, Mann-Whitney U) will be performed.

Setting the pilot trial sample size in order to minimize overall size has been based on the Kieser and Wassmer approach. They applied the 80% upper control limit approach to sample size calculation and found that a pilot trial sample size between 20 and 40 would minimize the overall sample size for a main study sample size of 80-250, corresponding to standardized effect sizes of 0.4 and 0.7 (for 90% power based on a standard sample size calculation) [31].

All analyses will be carried out using the Statistical Package for Social Sciences, version 19. A 95% confidence interval will be used for all analyses.

### Ethics

Research procedures in this pilot study complied with European Union and national legislation (ie, the Charter of Fundamental Rights of the EU, Directive 95/46/EC of the European Parliament, and of the Council of October 24, 1995, on the protection of individuals with regard to processing of personal data and the free movement of such data). PRECIOUS partners respect the latest Helsinki Declaration and follow the ethical guidelines provided by their national scientific societies and their local research institutions. This pilot study and all studies included in PRECIOUS have been presented to the hospital research ethics committee for approval and have been accepted (reference PR(AG)212/2014). All participants are adult volunteers, and informed consent has been obtained in all cases. None of the methodologies and technologies used are known to inflict any physiological or psychological damage on participants. The investigations included in the project are not medical examinations. This study has been presented to the University Hospital Vall d’Hebron research ethics committee for approval, and all issues resolved satisfactorily.

The first stages of participant recruitment (already performed) were carried out in the outpatient clinic of the Endocrinology Department of Vall d’Hebron Research Institute and Psychiatry department (ie, the bariatric surgery consultation unit). All individuals interested in taking part were contacted via telephone and introduced to the general nature (eg, purpose, methods) of the study. An informed consent was obtained in a face-to-face interview before starting the study. It included a written description of the study using language and terminology that is reasonably understandable to the participants (eg, all measurement techniques and procedures). The participants were also told that they may withdraw from the study at any time without consequences of any kind. In addition, the relevant details of data protection and storage were described to them.

Personal data are anonymized and made inaccessible to third parties. All questionnaires (with no participant name) completed by the participants are stored in a dedicated locked room. The digital identification data file containing participant names and contact information is stored in a safe. When analyzing data, researchers used digital data files with no identification data (a participant number will be used for data-linkage purposes). Only the researchers involved in the project have access to these data files. At the end of the 3-year project (or earlier), all questionnaires and identification data files will be destroyed. That is, the remaining digital data files will be such that the participants (data subjects) can no longer be identified.

## Results

This pilot study has concluded with the inclusion of 31 morbidly obese patients. The anticipated completion of data analysis and dissemination of final results is June 2017.

## Discussion

### Principal Considerations

The main goal of this pilot study is to assess users’ overall satisfaction, usability, and acceptability of the PRECIOUS system and to explore if MI is a feasible solution to foster adherence to the PRECIOUS system in a sample of end users. Moreover, we explore if PRECIOUS triggers behavior change and builds motivation to maintain sustained change towards healthy lifestyles.

The use of mobile computing and communication technologies in health care and public health is continuously expanding and evolving [32]. A report from the IMS Institute for Healthcare Informatics has shown that more than 165,000 mHealth apps are now available on the market, more than doubling over the past 2 years [1,2]. A high percentage of these apps are focused on healthy lifestyles, providing an attractive offer with different activities and incentives aimed at catching people’s attention [8,9,33-35]. Physical activity, healthy diet, and stress management are star products among this huge offering of apps. The World Health Organization discusses the benefits of using ICT in health care settings in terms of better access to information, improved communication between colleagues and patients with health providers, facilitating continuing professional development, and providing learning tools for health care professionals, patients, and the community as a whole. This huge market holds promising benefits, especially for users. A recent report from the European Union assessed the socioeconomic impact of mHealth solutions and found that they offer the potential of a cost saving of €76 billion, with the technology helping 54 million patients avoid the risk of developing a lifestyle disorder [32]. However, 75% of apps are abandoned just 3 months after being downloaded. A possible explanation for such low rates of adherence might be the initial design of the apps. A vast number of apps already exist for different health conditions, but the majority offer similar functions and fail to include, from the very initial phases of service design, a comprehensive motivational framework and sufficient psychological parameters to ensure engagement and mid- to long-term adherence to the service. PRECIOUS is intended to overcome such limitations and aims to combine a multidisciplinary scientific corpus of knowledge, nurtured from ICT, engineering, psychology, and mental health sciences. Available evidence-based MI interventions showing positive results will also serve as a reference. Thus, the PRECIOUS system integration, verification, and validation are guided by the following primary objectives: (1) to ensure that the PRECIOUS system is free from defects and acceptable for use, and (2) to verify that the PRECIOUS system is able to fulfill the requirements.

mHealth apps with motivational elements provide psychological incentives for users to participate by appealing to their sense of achievement and enjoyment. A reminder alone may not be a compelling enough reason to, for instance, go for a run, keep a healthy diet, or take one’s medication over time. Yet, when given rewards for complying, users have been shown to participate at a higher rate. The best approach to ensure sustained motivation is to build a solid motivational framework and also be able to discern the differences of potential users and to treat them differently [36]. PRECIOUS takes into account all these factors and adapts to users by generating a virtual individual model by asking them for their outcome goals from the very beginning and by remembering in a personalized manner progress, achievements, and meaning of actions. In this sense, users will establish their outcome goals from the start and these will be linked to health behaviors (eg, physical activity, healthy diet, stress). PRECIOUS will meet their needs by offering them different apps to guide them to success. Thus, the comprehensive motivational framework of PRECIOUS puts the user in the center of the action and promotes adherence, empowerment, and health self-management by creating a positive journey for users. All of this is expected to promote and contribute to maintenance of long-term motivation for behavior change.

In brief, interventions that merge up-to-date cognitive-behavioral science and MI with interactive technology may be an efficient and innovative way to address some of these issues because they can be disseminated to new settings, populations, and areas that might not otherwise have the capacity for in-person evidence-based care. MI delivered by new technologies (eg, mobile apps) can address these issues because the content is programmable, automated, and personalized, which may be particularly important when disseminating MI in diverse populations and in different languages. This approach is also less expensive than one-on-one treatment, offers easy access, and the anonymity overcomes the stigma sometimes associated with formal treatment [37].

### Limitations

Our study protocol has some drawbacks that should be taken into account. First, representativeness and thus, generalization of preliminary findings are likely to be an issue, specifically, due to the nature of the sample (ie, a nonprobabilistic convenience sample) and because we will limit the pilot test to users with Android operating systems. It may be possible that users with iOS devices have a different profile and motivations towards healthy lifestyles and behavior change. To understand this limitation, the PRECIOUS project consortium plans to perform future trials with users of iOS. Second, the PRECIOUS app will experience some updates within the scope of the trial. Considering that app schedule updates are not fully customizable, it is possible that patients will not have exactly the same experience since availability of some features could vary. Additionally, assessment sessions and the data collection process are planned to be long because more than 150 items are expected to be collected and commented on. This might reduce the validity and accuracy of the answers. Thus, future studies should focus on the most relevant aspects to reduce bias in the data collection process and reporting. Finally, technological literacy and familiarity with eHealth solutions should also be considered a potential limitation. The literature on this topic lacks empirical research on the social practice of ICT and possible social/health exclusions if these solutions were implemented on a large scale. Research should enable us to extend our understanding of the barriers to adoption and integration of ICT in the health context and with specific sample populations. However, in this protocol study we devoted the initial assessment session to explain the eHealth solution. This was reinforced by follow-ups (telephone and face-to-face).

### Conclusions

The PRECIOUS project will provide new innovations in preventive health care that include (1) a new automated service that analyzes user health to identify present and future risk factors, (2) a novel motivational system that boosts the required user actions to reduce unhealthy habits and promote healthy ones, and (3) an innovative gamified user interface, including key motivation elements from the gaming industry to trigger and maintain behavioral change. Research on the health-specific effects of this solution on users must be carried out, as well as the economic impact on the health care system that such a preventive system could have.

## Abbreviations

FP7: 7th Framework Programme for Research and Technological Development
ICT: information and communication technologies
MI: motivational Interviewing
PRECIOUS: PREventive Care Infrastructure based On Ubiquitous Sensing

## Appendix

Multimedia Appendix 1 Evaluation report fro PRECIOUS project.
