# Supplementary material for: PREventive Care Infrastructure based On Ubiquitous Sensing (PRECIOUS): A Study Protocol
Source: JMIR Res Protoc. 2017 May 31;6(5):e105. doi: 10.2196/resprot.6973 (PMC5471342; doi:10.2196/resprot.6973)
Supplement: Multimedia Appendix 1 [file resprot_v6i5e105_app1.pdf]

Evaluation Summary Report

Proposal : 611366
Acronym : PRECIOUS
Program : FP7
Call : FP7-ICT-2013-10
Funding scheme : Small or medium-scale focused research project -STREP - CP-FP-INFSO
Duration : 36 months
Activity : ICT-10-5.1 - Personalised Health, active ageing and independent living

PRECIOUS
PREventive Care Infrastructure based On Ubiquitous Sensing

Proposal submitted by :

N°	Proposer name	Country	Total cost (€)	%	Grant requested (€)	%
1	AALTO-KORKEAKOULUSAATIO	Finland	848,383	23.05	684,339	24.36
2	HELSINGIN YLIOPISTO	Finland	459,894	12.50	344,920	12.28
3	Firstbeat Technologies Oy	Finland	493,120	13.40	369,840	13.17
4	Institut Mines-Telecom	France	503,102	13.67	377,326	13.43
5	CAMPDEN BRI	United Kingdom	383,600	10.42	287,700	10.24
6	UNIVERSITAET WIEN	Austria	705,452	19.17	530,214	18.87
7	INSTITUT CATALA DE LA SALUT	Spain	286,480	7.78	214,860	7.65
	**Total**		**3,680,031**	**100%**	**2,809,199**	**100%**

Abstract :
PRECIOUS: PREventive Care Infrastructure based On Ubiquitous Sensing will provide a preventive care system to promote healthy lifestyles, which is comprised of three components: (1) transparent sensors for monitoring user context and health indicators (food intake, sleep and activity) deliver ambient data about current user behavior; (2) users are represented by individual virtual models which allow inferring health risks and desired behavioral changes; (3) state-of-the-art motivational techniques (originating especially from gamification and motivational interview) trigger a set of feedback tools to change the user habits toward more healthy conduct. While related projects usually focus on developing specific sensors, middleware solutions, health monitoring systems, eHealth services, etc., both the individual virtual model and the associated motivational tools will now provide key innovation steps towards a preventive care system with measurable impact on user behavior and thus a clear potential for large scale commercialization and sustainable societal footprint (e.g. with respect to cost saving in the public health sector as well as life quality improvements). To reach these goals, PRECIOUS consortium gathers partners from academia, SMEs and hospitals with comprehensive expertise in networking, pervasive sensing, cognitive analysis, nutrition research, semantic technologies and motivational techniques. We have chosen to focus on type II diabetes as a central use case, while our prototype will be easily adaptable also to other lifestyle-induced diseases. The system will not only detect and communicate detailed early warning signs, but also provide forecasts of future developments and associated problems (if change recommendations are not followed). Extensive lab- and field-based user trials will demonstrate the efficacy of the PRECIOUS system and prove its positive and lasting impact on individual quality of life as well as public health sector development.

Evaluation :

**1.Scientific and/or technological excellence (relevant to the topics addressed by the call)** ***(Threshold 3.0/5 ; Weight 1.00)***	***Mark:***

The proposal addresses target 5.1.a. very well in all aspects.<br><br>The proposal is aiming to provide a preventive care system to promote healthy lifestyles, with a high quality sensing platform, virtual models, and a motivational system. The concept is very sound, very well structured, focussed and described, and its related objectives are very good.<br><br>The state of the art is profoundly described. The envisioned system will be based on state of the art technology in addition to new methodologies of motivational interviews, and it will build follow-up mechanisms with medical experts. Key innovations are related to both the individual virtual model and the associated motivational tools with measurable impact on user behaviour.<br><br>The theoretical framework is well elaborated and presented. The small scale pilot and field tests are well designed. However, the monitoring of the food intake is not described in sufficient detail.	4.50

**2.Quality and efficiency of the implementation and the management** ***(Threshold 3.0/5 ; Weight 1.00)***	***Mark:***
The management plan, structure and procedures are adequate.<br><br>The quality of individual participants is very good, and they have relevant experience.<br><br>The consortium is a well-balanced mixture of academic and research institutions, and SMEs, with complementary expertise.<br><br>The planned budget is well prepared and relevant. The allocation of resources is appropriate and justified.<br><br>The risk and contingency plan is excellent.	5.00

**3.Potential impact through the development, dissemination and use of project results** ***(Threshold 3.0/5 ; Weight 1.00)***	***Mark:***
The proposal will contribute to the expected impacts very well. Improving lifestyle changes will decrease the need for medical intervention, and could have a significant economic impact. Commercialization of the envisioned results could lead to organization of novel effective preventive care services with measurable impact on user behaviour.<br><br>The proposal has a general, yet adequate dissemination plan, which includes the specific dissemination plans of most participants.<br><br>The overall exploitation plan is realistic, but lacks some details related to the implementation of the service. An exploitation plan is proposed by most partners.<br><br>The management of intellectual property is adequately dealt with.	4.00

**4.Remarks** ***(Threshold 10.0/15)***	***TOTAL:***
	13.50

**Does this proposal have ethical issues that need further attention?** ***(If yes, please complete an ethical issues report form (EIR))***	N

For each criterion under examination, score values indicate the following assessments. Half point scores may be given :
0- *The proposal fails to address the criterion under examination or cannot be judged due to missing or incomplete information*
1- Poor. *The criterion is addressed in an inadequate manner, or there are serious inherent weaknesses.*
2- Fair. *While the proposal broadly addresses the criterion, there are significant weaknesses.*
3- Good. *The proposal addresses the criterion well, although improvements would be necessary.*
4- Very Good. *The proposal addresses the criterion very well, although certain improvements are still possible.*
5- Excellent. *The proposal successfully addresses all relevant aspects of the criterion in question. Any shortcomings are minor.*
